# Investigating the Correlations Between Weather Factors and Mycotoxin Contamination in Corn: Evidence from Long-Term Data

**DOI:** 10.3390/toxins17020077

**Published:** 2025-02-08

**Authors:** Alexander Platzer, Younos Cherkaoui, Barbara Novak, Gerd Schatzmayr

**Affiliations:** 1 dsm-firmenich, Animal Nutrition and Health R&D Center, Technopark 1, 3430 Tulln, Austria; barbara.novak@dsm-firmenich.com (B.N.); gerd.schatzmayr@dsm-firmenich.com (G.S.); 2 dsm-firmenich, DSM Nutritional Products Ltd., 4303 Kaiseraugst, Switzerland; younos.cherkaoui@dsm-firmenich.com

**Keywords:** mycotoxins, weather, climate change, deoxynivalenol, zearalenone, aflatoxins, ochratoxin A, fumonisins, T-2 toxin

## Abstract

Mycotoxins are secondary metabolites produced by certain fungi, posing significant health risks to humans and animals through contaminated food and feed. These fungi, and consequently the mycotoxins which they produce, are strongly influenced by weather, and this shifts over time due to climate change, leading to more frequent and severe events, such as heat waves, storms, and heavy rainfall. This study investigates how long-term weather trends and climatic factors impacted mycotoxin levels in corn samples over a 17-year period (2006–2022) across 12 countries, with a focus on 136 specific weather features. Among all potential relationships, we found *Aspergillus* toxins and fumonisins to be positively correlated with temperature, while deoxynivalenol and zearalenone are negatively correlated. Additionally, the dew point, particularly its 90th percentile value, is positively correlated with *Aspergillus* mycotoxins. We also identified significant patterns associated with wind direction. Collectively, these findings offer a comprehensive overview of mycotoxin–weather correlations, which may also be projected into future scenarios.

## 1. Introduction

Mycotoxins are secondary metabolites produced by fungi that often contaminate raw materials used for food and feed, posing significant health risks to both humans and animals. Their production is favored by specific weather conditions, which vary depending on the type of fungal strain. As a result, researchers have been investigating the impact of current climate change and extreme weather patterns on the occurrence and distribution of mycotoxins for over a decade [1,2,3].

For instance, *Aspergillus* species, which produce the highly toxic metabolite aflatoxin B1 (AFB_1_), prefer warmer conditions and grow best between 25 °C and 35 °C [4,5]. In contrast, *Fusarium* species, which produce the most common mycotoxin, deoxynivalenol (DON), thrive best at temperatures ranging from 20 °C to 30 °C [6,7].

It has been shown that the interaction of water availability and temperature affects the growth of *Aspergillus* strains, thereby influencing AFB_1_ production [8]. Medina’s research group demonstrated that a third environmental stress factor, namely elevated carbon dioxide levels, can enhance the gene expression responsible for AFB_1_ production [9]. Furthermore, high humidity and moisture levels are critical factors influencing the growth of fungi. The risk of mycotoxin contamination, especially for DON, can significantly increase, if wet conditions and high humidity occur particularly around flowering or harvest time. On the other hand, dry conditions and hot weather can also trigger AFB_1_ production by stressing the plants, making them more susceptible to fungal infections (reviewed by Daou et al. [10]; and Liu and van der Fels-Klerx [11]). In 2015, Salvacion et al. [12] assessed the risk of aflatoxins and fumonisins contamination on corn in the Philippines, as this crop is highly susceptible to those two mycotoxins [13]. They concluded that increasing temperature and rainfall favor fumonisins but limit aflatoxin contamination in the country [12]. Another research group extrapolated that the risk of aflatoxin will most likely increase in nearly 90% of all maize in the United States from 2031 to 2040 [14]. It is also described that elevated carbon dioxide concentrations, increased temperatures, and the interplay between drought and heavy rainfall particularly affect fungal growth and mycotoxin occurrence [15].

In addition to the well-studied weather conditions, such as temperature and humidity, other weather features, such as wind, can also correlate with mycotoxin occurrence. Wind can act as a vector for dispersing fungal spores over long distances, leading to contamination of crop plants in different areas [11]. Wind can also have an indirect effect by reducing humidity and increasing plant transpiration. This, in turn, can stress the plants and make them more susceptible to fungal infection [16].

The aim of our study was to investigate weather trends over 17 years and compare them with certain *Fusarium* and *Aspergillus* mycotoxins in corn samples. For our analysis, mycotoxin data from 12 countries were correlated with 136 different weather features. The countries Argentina, Austria, China, France, Germany, Hungary, Italy, Poland, South Africa, Spain, Turkey, and the United States were chosen based on the required number of corn samples to ensure statistical significance.

## 2. Results

### 2.1. Weather Trends

Before correlating weather data with mycotoxin occurrences, it was necessary to verify if significant weather trends or extreme events could be detected. To achieve this, we selected the period from 2006 to 2022, aligning with the time frame of our mycotoxin survey data. Then, we consolidated the available hourly weather data (2006–2022) around harvest time from seven meteorological variables into 136 aggregated weather features for each country and harvest year. For more details, see Materials and Methods.

**Figure 1:**
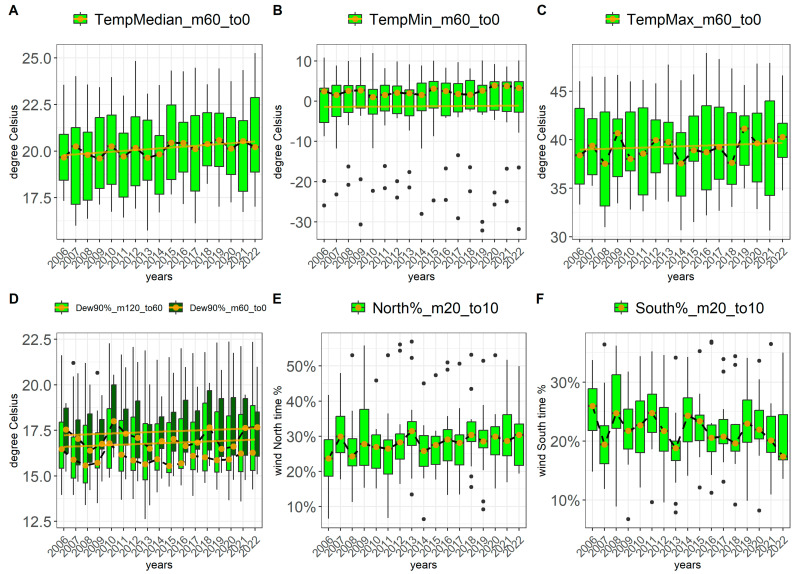
Weather trends from 2006 to 2022 (17 years). Boxplots per year show the values of different weather features for 12 countries—so exactly 12 values per year. Golden points and dashed lines indicate median values. The transparent orange line shows the weak trend (ordinary least squares). The following countries were included: Argentina, Austria, China, France, Germany, Hungary, Italy, Poland, South Africa, Spain, Turkey, and the United States. The weather features, represented on the y-axis, are specified in the title line of each plot. (**A**–**C**) represent the median, minimum, and maximum temperatures in the time frame of 60 days before harvest to harvest (m60_to0). (**D**) shows the averages of the 90th percentiles of the dew point for location nodes per country across two different periods. (**E**,**F**) show the ratios of time the wind comes from the north and south. (**D**–**F**) exhibit significant trends over these 17 years: the year-to-year absolute difference decreases. The string following the abbreviated weather feature name indicates the period, e.g., “m20_to10” denotes the time from 20 days before harvest to 10 days after harvest.

Although the primary effect of climate change is global warming [17], we did not observe any significant temperature trends over these 17 years within the specific time frames around harvest, except for the maximum temperature in Spain (Table S1). Despite the absence of significant temperature trends over this period, we examined all our weather features related to temperature in relation to mycotoxin levels. See Figure 1A–C for the trends of the minimum, maximum, and median temperatures over the years. No significant temperature trends around harvest were observed over these 17 years.

In the following text, the exact variable names are in parentheses, when referred to. See Section 4.2.1 Weather Features for details, and Table S2 for all weather feature labels.

Among the 136 weather features, the following showed significant changes over the 17 years: dew point and ratios of wind direction (AvgNodeDewpoint90Percentile, WinddirectionNorthRatio, WinddirectionSouthRatio). Additionally, the air pressure varied over the years. However, since pressure is strongly location-dependent, with greater variation between locations than within a location, we have not included air pressure in our analysis. The dew point (AvgNodeDewpoint90Percentile) is changing in all of the 12 surveyed countries within the 2-month and the 6-month period in the same way: the year-to-year absolute difference decreases. In the long term, the dew point is increasing due to climate change and is considered to be positively correlated with temperature [18].

Also, the wind direction shows a change in the shortest period around harvest (WinddirectionNorthRatio, WinddirectionSouthRatio, 30-day period): the year-to-year differences decrease.

Refer to Figure 1D,E for visualizations of dew point and wind direction over time (raw *p*-values: 5.56 × 10^−5^, 1.75 × 10^−5^, and 9.73 × 10^−5^, respectively). See Table S1 for all *p*-values of the potential trends of our 136 defined weather features.

### 2.2. Temperature and Mycotoxins

Among the 136 weather features analyzed in relation to mycotoxin levels at the corresponding harvest, we observed that *Aspergillus* mycotoxins and fumonisins were positively correlated with median temperature, while deoxynivalenol (DON) and zearalenone (ZEN) were negatively correlated with temperature. For *Aspergillus* mycotoxins and fumonisins, there appears to be a critical threshold at approximately 22 °C, where a notable increase in concentration occurs. In contrast, for DON and ZEN, no specific temperature threshold was evident; instead, their relationships with temperature are characterized by a more continuous correlation.

**Figure 2:**
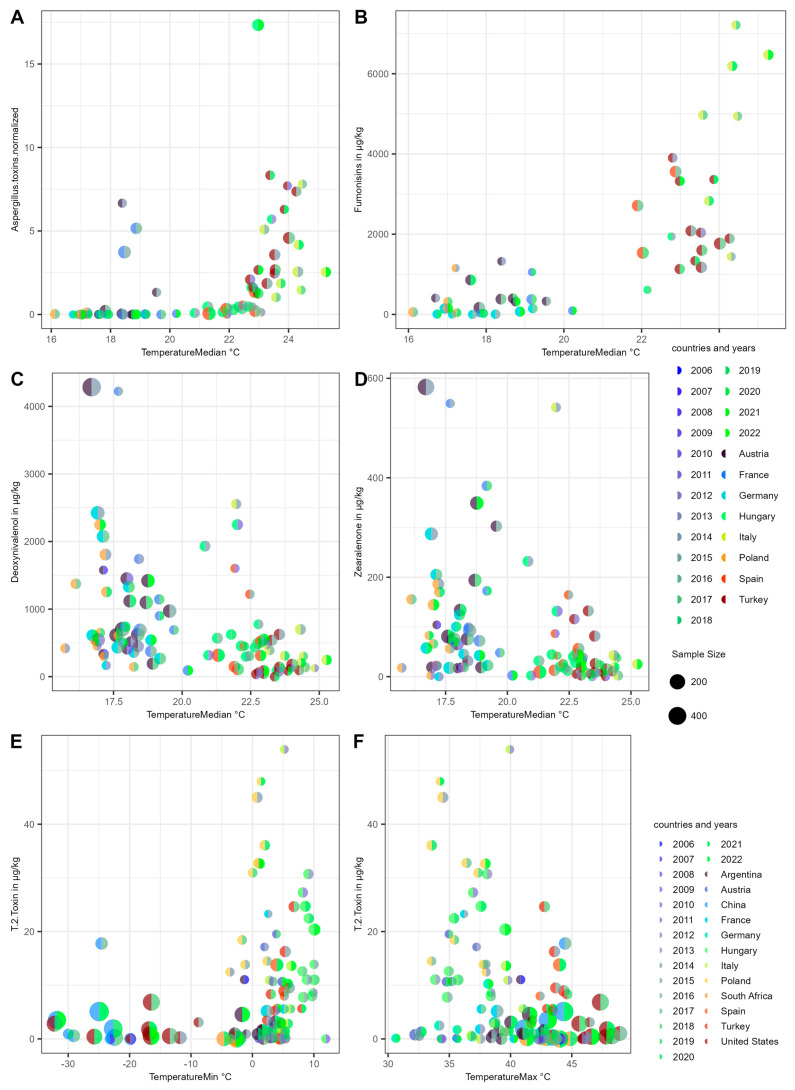
Scatter plots of temperature and mycotoxins. (**A**–**D**) Median temperature correlates with different mycotoxins: *Aspergillus* toxins (aflatoxins, ochratoxin A), fumonisins, deoxynivalenol, and zearalenone. Positive correlations in (**A**,**B**) and negative correlations in (**C**,**D**) are clearly visible. The correlations of other temperature weather features for these 4 mycotoxins (groups) are going in the same direction, and there is no significant opposite—see text and Table S3 for more information. The upper legend on the right pertains to (**A**–**D**), and the lower legend pertains to (**E**,**F**). The sizes of the (semi-) circles are scaled equally in all 6 diagrams. (**E**,**F**) The T-2 toxin abundance is positively correlated with minimum temperature and negatively correlated with maximum temperature. The lower legend on the right pertains to (**E**,**F**). The scale for sample sizes is the same for all diagrams; the scale is linear for size, but 0 corresponds to a certain size, not to invisibility. The minimal sample size is 5 by filtering. The colors for each point in the diagram depict its country on the left and the year on the right. The weather data correspond to the period from 60 days before harvest until harvest. The FDR-corrected *p*-values of Spearman correlation coefficients for (**A**–**F**) are as follows: 9.01 × 10^−11^, 5.98 × 10^−9^, 7.45 × 10^−6^, 0.004, 0.024, and 0.01.

For fumonisins, the average concentration at median temperatures below 22 °C is approximately 400 μg/kg, whereas at temperatures above 22 °C, the average concentration increases to about 3000 μg/kg. This trend is consistent across European countries and remains similar across the 12 countries analyzed (see Figure 2A–D). Although the median temperature was chosen for simplicity, these significant correlations are observed across most temperature-related features and throughout all three selected time periods. No temperature feature showed a significant correlation in the opposite direction from the majority, although not all temperature features were significantly correlated with mycotoxins. Table S3 provides a comprehensive overview of all feature combinations.

An exception is the T-2 toxin, which does not correlate consistently with all temperature features. Specifically, the minimum temperature is positively correlated with T-2 toxin concentrations, while the maximum temperature is negatively correlated (see Figure 2E,F). The highest concentration of T-2 toxin of 53.9 μg/kg was detected in Italy in 2012, with corresponding minimum, maximum, and median temperatures of 5.2 °C, 40 °C, and 24.8 °C, respectively. When minimum temperatures decrease to −5 °C, T-2 toxin concentrations generally drop below 5 μg/kg. Similarly, when maximum temperatures rise to 45 °C, T-2 toxin levels also fall below 5 μg/kg (see Figure 2E,F).

### 2.3. Weather Trends and Mycotoxins

**Figure 3:**
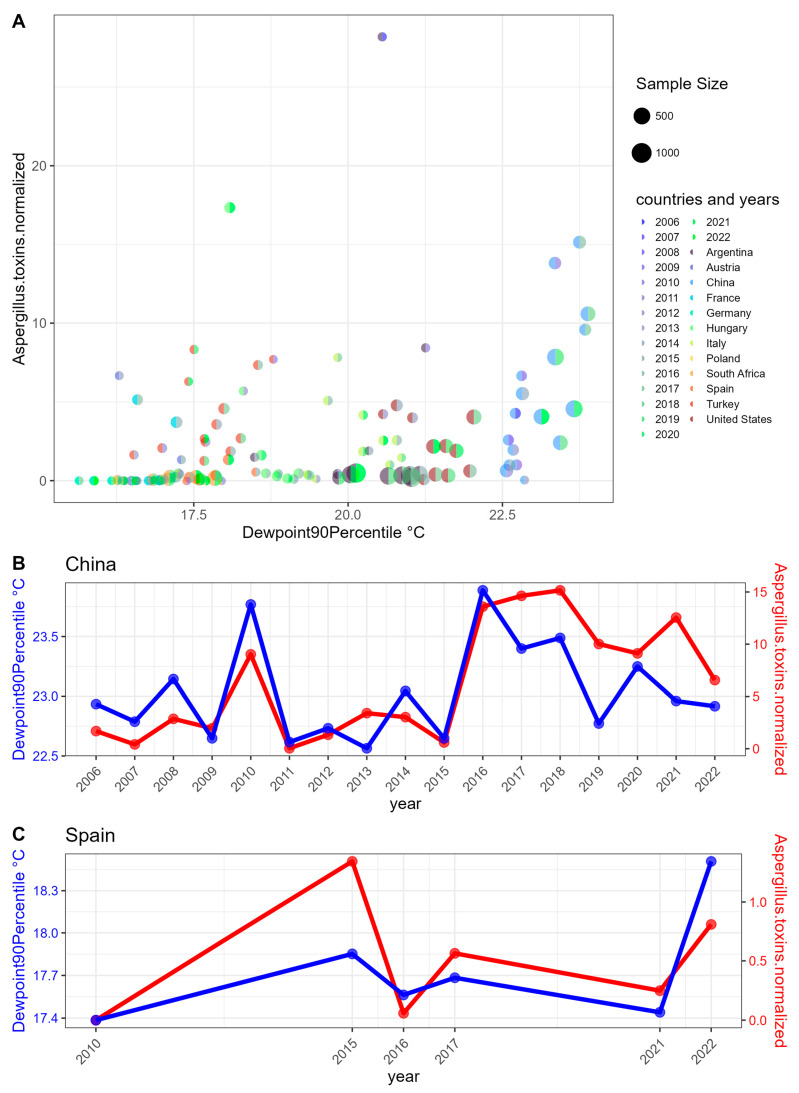
Relationship between dew point and *Aspergillus* toxins normalized. “Dewpoint90Percentile” represents the 90th percentile value of dew points in each country during the period from 60 days before harvest until harvest. (**A**) Scatter plot. The minimal sample size is 5 by filtering. The colors for each point in the diagram depict its country on the left side and the year on the right side. (**B**,**C**) China and Spain as line charts, as these two countries particularly drive the correlation. However, the relationship between dew points and *Aspergillus* toxins is not significant for any individual country, primarily due to sample size. The Spearman correlation is significant only when considering all countries together (FDR-corrected *p*-value: 6.87 × 10^−9^).

Additionally, we focused on examining the intersection of significant correlations of mycotoxin levels and the 136 defined weather features that demonstrated significant trends over the 17-year study period. Among these features, the 90th percentile of the dew point (Dewpoint90Percentile) exhibited a positive correlation with *Aspergillus* mycotoxins during the period from 60 days before harvest until harvest (see Figure 3A). This correlation appears to be primarily driven by data from China, where the Dewpoint90Percentile starts at 22.5 °C (Figure 3B). Despite the visually apparent pattern, the values from China alone are only statistically borderline significant (with an FDR-corrected *p*-value of 0.05), and there is no significant trend of the dew point in China over the 17-year period. This lack of statistical significance is most likely due to the small sample size. Spain presents another interesting case in this context, with an even smaller sample size (Figure 3C).

Over the short period (20 days before harvest to 10 days after harvest), no significant correlation with the dew point was observed. However, over the long period (6 months), the correlations differed.

**Figure 4:**
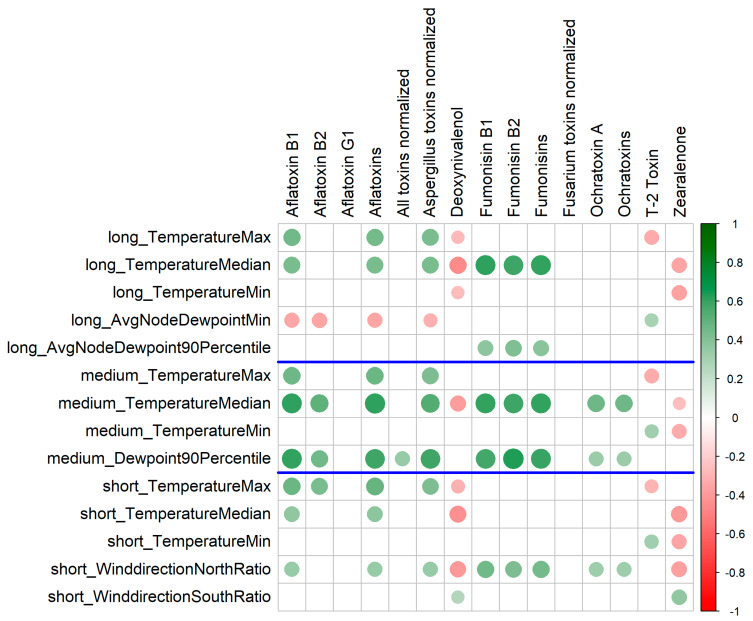
Correlations between mycotoxin concentrations and weather features. The FDR-significant, “most covering” correlations are shown from all pairwise calculations; only statistically significant results are depicted. The labels “long”, “medium”, and “short” indicate time intervals, as defined in Section 4.2.1. For a complete list of correlations, refer to Table S3.

Figure 4 presents the FDR-significant “most covering” correlations between mycotoxin concentrations and weather features. The term “most covering” indicates that among several weather features with high intercorrelations, only one representative feature is shown. Additionally, only the correlations observed across all countries combined are depicted. All correlations mentioned in the main text are included. For a complete list of correlations between weather features and mycotoxins, refer to Table S3.

**Figure 5:**
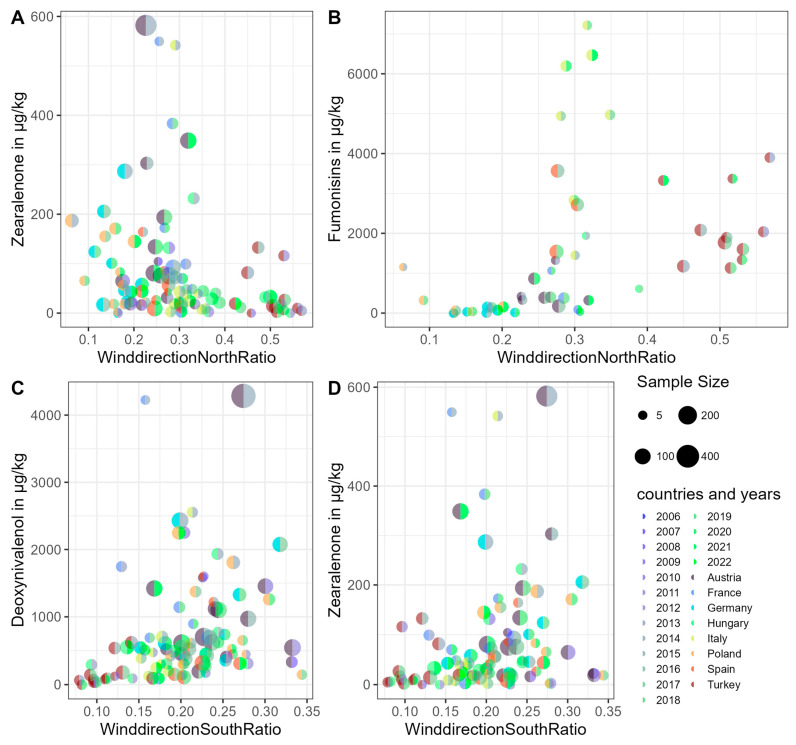
Scatter plots of wind direction and mycotoxins (**A**–**D**). “WinddirectionNorthRatio” represents the proportion of time the wind originates from the north in each country during the period spanning 20 days before harvest to 10 days after harvest, while “WinddirectionSouthRatio” reflects the proportion of time the wind originates from the south. Each point is colored by country (left side) and year (right side). The minimal sample size was set to five after filtering. FDR-corrected *p*-values are 0.011, 2.8 × 10^−5^, 0.001, and 0.07. The last correlation is not significant when considering only Europe—shown here for consistency—and an alpha level of 0.05, but is significant across all countries combined (FDR-corrected *p* = 0.0005).

Wind direction is another meteorological feature that demonstrates a two-fold significance, showing both a notable trend over the 17-year period and a significant correlation with certain mycotoxins. Specifically, *Aspergillus* mycotoxins and fumonisins exhibit a positive correlation with the proportion of time the wind blows from the north (WinddirectionNorthRatio). Conversely, DON and ZEN show a negative correlation with the WinddirectionNorthRatio and a positive correlation with the proportion of south wind (WinddirectionSouthRatio)—see Figure 5 for details. In the case of fumonisins, a threshold appears to exist around 20% of north wind occurrence: below this threshold, fumonisin concentrations typically remain under 1000 μg/kg, while above this level, the majority of concentrations exceed 1000 μg/kg. The other correlations (*Aspergillus* mycotoxins, DON, ZEN) appear to be more variable but generally exhibit continuous patterns (see Figure 5 for fumonisins and ZEN, with additional information in Table S3 for all correlations and their significance).

### 2.4. Confounders/Dependencies of Wind Directions

Beyond the direct correlations, potential confounders should also be considered. Many potential confounders are possible, including the weather variables themselves—any weather feature may be confounded by other weather features—and the location, for which we have the country information. One motivation for this investigation is the controversial relationship between mycotoxins and wind direction. While Figure 5 appears sound, and the *p*-values are clearly significant, country-level analyses present a different picture (Figures S1 and S2). The overall negative relationship between north wind and zearalenone (Figure S1) is apparent in some countries (e.g., France, Spain, China, and Turkey), whereas in others, they appear unrelated. Notably, no single country exhibits a statistically significant relationship, likely due to a limited number of data pairs; significance emerges only when considering all countries collectively. A similar pattern is observed for the relationship between north wind and fumonisins—clearly evident in some countries but not in others—yet, it is not merely confounded by country. For detailed per-country diagrams, see Figures S1–S6.

**Figure 6:**
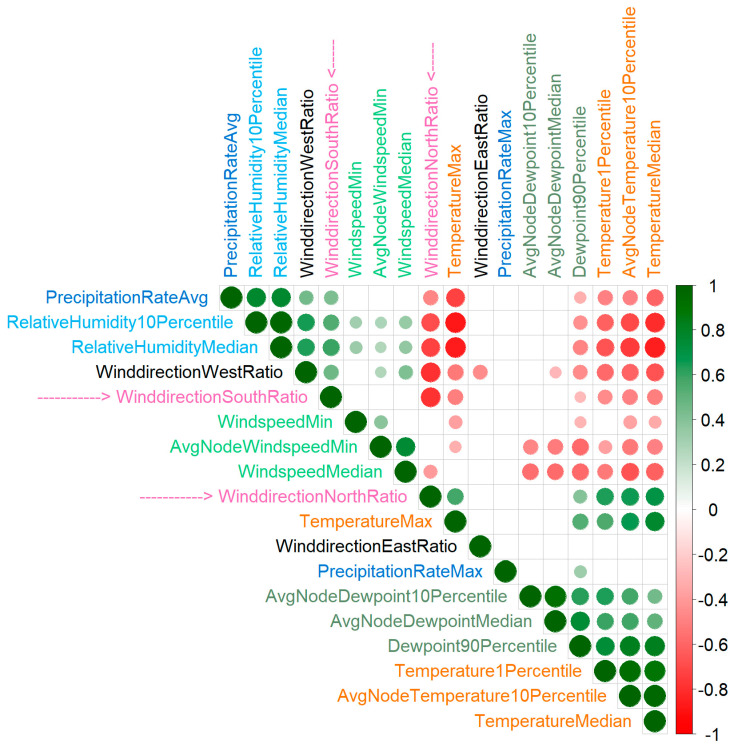
Correlations between weather features. One representative feature is taken for each strongly correlated weather feature cluster. All pairwise correlations were calculated. The time period is from 20 days before harvest to 10 days after harvest. Only significant correlations are shown (Bonferroni-corrected). The label colors are used for grouping by type: one color for temperature-related features, another color for precipitation, etc. The labels WinddirectionSouthRatio and WinddirectionNorthRatio are more prominent because these are most discussed in the main text.

To effectively interpret wind directions, it is essential to examine their correlations with other weather features. Although the correlations among all 136 weather features may appear complex (see Figure S7), several clusters emerge, where certain features exhibit strong correlation with each other. These features can be reduced to one representative per cluster (Figure 6). Expected correlations include precipitation positively correlating with relative humidity and temperature positively correlating with dew point. The latter is expected, as the dew point is tightly related to the absolute humidity, and warmer air has a higher capacity to retain moisture. However, there are also unexpected correlations, particularly with wind direction. For instance, the ratio of north wind is negatively correlated with wind speed, precipitation, and humidity, but positively with temperature. Conversely, the relative duration of south wind is negatively correlated with temperature. It is important to note that these correlations are between aggregated annual values, specifically within the period around the harvest. Thus, the correlation is between the fraction of time when wind originates from the north or south and, e.g., the median temperature during this period, rather than the average temperature at the time the wind is coming from the north.

## 3. Discussion

### 3.1. Our Results and Other Studies

Our analysis, based on a comprehensive dataset, reveals several patterns linking mycotoxin production to long-term weather conditions. Some of these correlations—particularly those involving temperature—have been previously reported by others, albeit primarily for specific regions. For instance, it has been published that “with increased global warming more aflatoxin contamination is expected in the Mediterranean basin” [10]; see also the extensive review of Paterson and Lima [19]. Additionally, previous studies have indicated that the optimal temperature range for T-2 toxin production lies between 10 and 15 °C [20], which aligns consistently with the patterns observed in our own data.

Dew point, a parameter closely associated with temperature, was also demonstrated to be of significant relevance in our study. Our results align with previous research indicating a positive correlation between water activity (which correlates with dew point) and ochratoxin A (OTA) production [21]. This is further supported by the generally high water activity levels required for the growth of *Aspergillus* species [10], which are known producers of mycotoxins such as aflatoxins and ochratoxins.

Regarding the observed correlation between mycotoxin levels and wind direction, a few studies have reported similar associations, although these are limited to one or two specific regions. However, direct comparisons with our results are challenging, as these studies typically use wind direction in degrees for correlation analysis [22,23].

### 3.2. Weather Time Periods

The timing of harvest is evidently a critical factor for mycotoxin contamination, as environmental factors prior to this point significantly influence mold growth and mycotoxin production. While weather conditions before harvest impact these factors, weather conditions after harvest should not be a concern if the crops are stored properly. However, the exact period before harvest that is most critical for mycotoxin contamination remains uncertain. While some studies suggest that the critical period ranges from 30 to 60 days prior to harvest [24], there is no definitive consensus. Based on this, we selected a 60-day window prior to harvest as one of our time periods for analysis.

To provide a comparative framework, we also included two additional periods: a longer time frame spanning from 120 days before harvest to 60 days after harvest, and a shorter period from 20 days before harvest to 10 days after harvest. While including time frames that extend beyond the harvest day might seem counterintuitive, it is worth investigating, as we have only one average harvest day per country and not a harvest day per sample. So, some samples are also harvested after the average harvest date.

### 3.3. Mycotoxin Data Characteristics and Filtering

Although our mycotoxin survey includes a variety of crops [25], we primarily focused on mycotoxins in corn samples due to the larger sample size available for this crop. We also examined results from other crops, such as wheat, but we observed that the less significant results were most likely due to smaller sample sizes. Presenting these two sets of results side by side could be misleading. Therefore, only corn-related results are included in this paper.

The majority of our samples provide only the country of origin and the sampling date, with no information on the harvest date. However, we do have data on the storage conditions, which allows us to be reasonably certain about which samples correspond to the most recent harvest. Given these limitations, we aggregated the mycotoxin data by country and year, which required the weather data to be aggregated similarly. Naturally, the aggregated mycotoxin concentrations exhibit varying levels of statistical variance, depending on the sample size (e.g., one sample versus 100 samples). To mitigate the influence of noise, we applied a threshold of at least five samples per country–year aggregation. This threshold was chosen as a trade-off; we experimented with different thresholds and found that lower thresholds resulted in highly increased noise, while higher thresholds led to the exclusion of many years of data.

### 3.4. Added-Up Mycotoxin Values

When correlating individual mycotoxins with weather features, we considered combining mycotoxins into their respective groups (e.g., Trichothecenes: deoxynivalenol, T-2 toxin) or combining all mycotoxins together. However, simply summing the concentrations would result in the mycotoxin with the highest average concentration dominating the total. Therefore, normalization of the mycotoxin concentrations was deemed necessary. After considering various options, we chose to normalize the mycotoxins based on their thresholds for detrimental effects (see Section 4.2.3 Materials and Methods for details). Although other equally valid normalization methods exist, our chosen approach offers greater interpretability as the thresholds for detrimental effects have meaningful relevance. Moreover, by applying this normalization, we obtained significant correlations with weather features that differ from those observed for individual mycotoxins.

### 3.5. Combined

Based on the observed correlations and associations, several long-term trends can be identified. Due to global warming—which is projected to increase temperatures by 0.2–0.3 °C per decade [26]—the levels of *Aspergillus* toxins and fumonisins are expected to rise, particularly when the median temperature exceeds 22 °C during the critical period from 60 days before harvest until harvest. It should be noted that we did not investigate how shifts in harvest time influence these outcomes. Such an investigation would require information on the harvest date for each sample.

The levels of deoxynivalenol and zearalenone decrease at elevated temperatures or shift northward to regions with temperatures within their optimal range. Such relationships between mycotoxin occurrence and temperature have also been reported in other studies [27,28]. Our research supports these associations in more detail and with a larger dataset.

Over the 17-year period from 2006 to 2022, we observed a slight upward trend in the dew point, similar to the trend for temperature (see Figure 1); however, none of these trends are statistically significant. Additionally, the dew point is strongly correlated with temperature (Figure 6). The significant pattern observed during this period is a decrease in year-to-year variability during the 60-day period prior to harvest. Furthermore, we found a positive correlation between the dew point and *Aspergillus* toxin levels. Based on these observations, we predict that *Aspergillus* toxin levels will increase with rising temperature and dew point, while exhibiting reduced fluctuation from year to year.

An intriguing element is the positive association between the ratio of wind originating from the north and *Aspergillus* toxins and fumonisins. Several factors could underlie this correlation. First, the direction of prevailing winds can influence regional microclimates, such as bringing cooler nights that might still maintain enough humidity for fungal sporulation. Alternatively, northerly winds could be carrying fungal spores or influencing the dispersal of spore populations. It was already described in other studies that wind plays a crucial role in fungal spore dispersal [29,30]. However, Parry et al. found that macroconidia are dispersed more easily by splash rather than by wind. Such wind patterns might also reflect broader climatic or geographic zones that have historically experienced higher fungal pressure. Clarifying the exact mechanisms would require additional investigation into how wind trajectories intersect with local environmental conditions (e.g., moisture retention, crop canopy, or microclimate shifts).

The negative correlations of temperature with DON and ZEN, as well as the negative correlation between the ratio of wind from the north and these toxins, may point to a different ecological niche preferred by *Fusarium* species responsible for these specific mycotoxins, such as *F. graminearum and F. culmorum*. Both species prefer high humidity (above 80%) and cool to moderate temperatures for optimal growth, which might be influenced by the correlated weather features [31,32].

The signs of our inferred correlations between wind directions and mycotoxins are not relevant for the long-term perspective, as the prevailing trend in the wind direction ratios indicates a decrease in year-to-year variability. This implies that these wind patterns may play a role in reducing the annual fluctuations in the levels of these mycotoxins.

Overall, these correlations highlight the complex interplay between fungal ecology and weather conditions, suggesting that mycotoxin production can vary widely depending on localized climatic factors. Understanding the relative contributions of temperature, humidity, and wind patterns to fungal colonization and mycotoxin biosynthesis is essential for anticipating contamination risks and developing timely mitigation strategies. Future work should investigate the combined effects of multiple environmental parameters—particularly under changing climate scenarios—and incorporate longer-term datasets to validate these findings and enhance predictive models for mycotoxin occurrence.

## 4. Materials and Methods

### 4.1. Raw Data

#### 4.1.1. Sample Collection and Mycotoxin Analysis

A total of about 27,000 corn samples, collected from 12 countries between June 2006 and January 2023, were analyzed to estimate mycotoxin concentrations. The samples, provided by the dsm-firmenich Mycotoxin Survey (all authors are working there), were examined using multi-toxin Liquid Chromatography with tandem mass spectrometry (LC-MS/MS), High-Performance Liquid Chromatography (HPLC), or Enzyme-Linked Immunosorbent Assay (ELISA) methods. Detailed information on the methods and laboratories involved can be found in Table 1.

Sampling, milling of samples, and homogenization of samples were performed as previously described [33]. In brief, samples were collected either after instruction or by a trained staff and a minimum of 500 g of sample was sent to one of the listed laboratories. To prevent humidity, samples were stored in paper bags or ventilated. Samples with high moisture content were dried prior to analysis. Once received, samples were promptly milled, homogenized, and finally analyzed with the respective method.

To ensure relevance of the data to freshly harvested crops, only these corn samples were included, excluding long-stored samples such as silages. Countries were selected based on the availability of sufficient data—specifically those with at least five samples per year and data spanning a minimum of five years between 2006 and 2022. The countries meeting these criteria were Argentina, Austria, China, France, Germany, Hungary, Italy, Poland, South Africa, Spain, Turkey, and the United States. This study focused on regulated mycotoxins or those with established guidance levels (see Table 1).

#### 4.1.2. Weather Data

The ERA5 [34] reanalysis dataset, produced by the European Centre for Medium-Range Weather Forecasts (ECMWF), provides comprehensive global weather and climate data by assimilating vast amounts of observational data into a consistent model. Spanning from 1950 to present, ERA5 delivers hourly data with high spatial resolution (31 km grid spacing) and incorporates over 250 atmospheric, oceanic, and land-surface variables. This dataset is widely used in research due to its ability to provide accurate and detailed historical reconstructions of weather patterns and climate variability. ERA5 has become a critical resource for climate research, impact assessments, and model validation, offering insights into both past and contemporary meteorological conditions. Hence, the ERA5 dataset was chosen as the source for generating the weather features used in climate change assessment and for correlation analysis with mycotoxin occurrences.

Known or assumed to be correlated with mycotoxin occurrences, the meteorological variables shown in Table 2 were used to generate weather features. These meteorological variables were computed from the original ERA5 variables [35]; see Table 3 for the dependencies. These variables are commonly known, except perhaps the dew point and components of wind. “The dew point of a given body of air is the temperature to which it must be cooled to become saturated with water vapor” [36]. Additionally, the horizontal wind is often decomposed into two perpendicular components—the *u*-component (eastward) and the *v*-component (northward)—representing orthogonal velocity components in a two-dimensional (or Cartesian) coordinate system [37]. Finally, the number before the *m* letter in the ERA5 variable name refers to the altitude (in meters) above the land surface at which the parameter is estimated. For example, the dew point temperature parameter used in this research is estimated 2 m above the surface of land. In processing meteorological data from ERA5, several transformations were applied to align variables with units commonly used in climatological analyses. Temperature and temperature dew point values, initially provided in Kelvin, were converted to degrees Celsius to facilitate interpretation and consistency. Similarly, surface pressure data were converted from pascals (Pa) to millibars (mb) to match standard meteorological conventions. Precipitation rates, originally in meters, were converted to millimeters to reflect rainfall measurements more accurately. Relative humidity was not directly available from ERA5 and was therefore approximated using the Magnus approximation, as described by Earth Science Stack Exchange [38]. Wind speed and direction (polar coordinate system) were derived from ERA5 wind components (Cartesian coordinate system) and then converted from meters per second (m/s) to kilometers per hour (km/h), following the procedures outlined in the ECMWF documentation [39]. These adjustments ensured that all meteorological variables were consistent and suitable for examining climate-related impacts on mycotoxin occurrence.

### 4.2. Data Filtering and Processing

#### 4.2.1. Weather Features

Hourly weather data were aggregated along two key dimensions to facilitate alignment with mycotoxin observations: (1) temporally, from hourly to yearly values, and (2) spatially, from 31 km resolution weather nodes to a single representative value per country. This aggregation was essential to ensure that the number of weather data points matched the available mycotoxin data (one averaged value per year per country) for statistical analysis. Additionally, for each weather node and year, a specific time window was applied based on the day of the year associated with corn harvest, as defined in our phenology database. Three distinct time frames were used to capture the critical weather period relative to harvest: (1) from 60 days before harvest to harvest day, (2) from 20 days before harvest to 10 days after, and (3) from 120 days before harvest to 60 days after. These time frames, present in Table 4, were selected to assess how varying pre- and post-harvest weather influences mycotoxin levels.

The weather features were calculated for each country and harvest year using hourly data from the 7 meteorological variables mentioned in Table 2. This resulted in a total of 136 weather features. For each period of the three previously mentioned pre-harvest to post-harvest periods, the hourly data were spatially averaged across weather nodes within a country. Key metrics such as the average, standard deviation, percentiles (1st, 10th, 90th, 99th), and extremes (minimum, maximum) were derived from variables like temperature, humidity, precipitation, and wind speed components.

Special handling was applied to directional variables and accumulated precipitation. Wind direction was categorized into directional ratios (e.g., north, east, south, west), and precipitation included metrics for extreme accumulations (3, 6, 24, and 120 h periods) to capture potential flood risks. Additionally, metrics were calculated for extended precipitation and drought periods, along with Cold and Heat Wave Magnitude Indices to assess extended cold or hot weather events. These aggregated features were designed to reflect both typical and extreme weather conditions that are relevant to mycotoxin occurrences in crops.

#### 4.2.2. Mycotoxin Survey Filtering

The 136 previously described weather features were generated annually for each country, with each year representing a specific crop harvest year. Consequently, the mycotoxin analytical data also needed to be aligned with the designated harvest year. To maximize sample availability, all samples collected around harvest time were included. Specifically, a time frame from 15 days before the start of harvest to 250 days after the end of harvest was selected for each country. To have a valid harvest year for a given country, at least 5 samples tested on the relevant mycotoxin had to be present per year; otherwise, the year was omitted in the analysis. Country with insufficient data, i.e., fewer than five samples per year and with more than one corn harvest period per year (e.g., Brazil), were excluded from the analysis. The countries meeting the requirements were as follows: Austria, France, Germany, Hungary, Italy, Poland, Spain, Turkey, South Africa, China, the United States, and Argentina, while the most commonly tested mycotoxins in these countries were as follows: aflatoxins B1, B2, G1, fumonisins B1, B2, deoxynivalenol, T-2 toxin, ochratoxin A, and zearalenone.

#### 4.2.3. Mycotoxin Features

Fourteen mycotoxin features were derived from the data of nine mycotoxins using four methods. The features were always averaged per year and country. The mycotoxin values were either taken as they were, summed up per mycotoxin group (e.g., aflatoxins), normalized by their threshold—which means divided by the threshold—and summed up per fungus group (e.g., *Aspergillus*), or normalized by their threshold and summed across all 9 mycotoxins. Table 5 lists the mycotoxins, their groups, and thresholds.

### 4.3. Merging Weather and Mycotoxin Features

As described in the weather features methodology, three distinct time frames were applied to filter and aggregate weather time series data (refer to Table 4). These time frames were aligned with the corn harvest period for each selected country, resulting in the generation of 136 weather values per country and harvest year. A similar approach was used for the mycotoxin features. Specifically, a single time frame was utilized to filter samples around the corn harvest period for each selected country and year (see Table 6). Consequently, up to 14 mycotoxin features were generated per country and harvest year, depending on the presence of at least five samples for that year. This alignment allowed the weather and mycotoxin features to be merged based on the corresponding country and harvest year.

### 4.4. Data Analysis

Unless explicitly stated otherwise, only significant relationships are mentioned in the results of Section 2.

#### 4.4.1. Correlations—Weather Trends

For all weather features, the Pearson and Spearman correlation between the feature and the year were calculated. For covering the trend of increasing (or decreasing) changes between years, the Spearman correlation of the years and the absolute difference in the weather feature between consecutive years was calculated. The minimum *p*-value of these three correlations per weather feature and region was taken to identify weather trends. As recognition of the multiple tests, an alpha level of 0.05/100 was used (a very loose correction; see Figure S7 for correlations within the weather features).

#### 4.4.2. Correlations—Weather Features to Mycotoxins

For all weather features and mycotoxin combinations, the Pearson and Spearman correlation was calculated. Because several plots indicated that Pearson correlation may often be misleading (due to its sensitivity to outliers), we used the *p*-value of the Spearman correlation to identify significant correlations. The *p*-values were corrected in a strict way using the Bonferroni method with α = 0.05, and more loosely using FDR with α = 0.05.

#### 4.4.3. Combined

In the analysis of significant weather trends, we focused on identifying notable weather features to mycotoxin combinations. The determination of significant weather trends utilized a single, loose threshold, as previously described. In contrast, for weather feature to mycotoxin combinations, we applied two levels of correction: a broad (FDR) correction and a stringent (Bonferroni) correction. These two levels of significance are also denoted in the tables with all correlations (Tables S1 and S3). Strict significance (Bonferroni) is denoted in dark green, and loose significance (FDR) is denoted in light green.

### 4.5. Visualization

For visualization and the major part of calculation, R software version 4.3.3 was used [40], and in R, the following special packages were used: ggplot2 3.5.0 [41], ggpubr 0.6.0 [42], and viridis 0.6.5 [43].

## Figures and Tables

**Table 1:** Mycotoxin analysis of corn kernel samples (only laboratories and methods with more than 50 samples).

Analyzer	Sample Number	Method	Ranges for Limits of Detection (µg/kg)										
			AF_tot_	AFB_1_	AFB_2_	AFG_1_	DON	FB_1_	FB_2_	FB_tot_	OTA	T-2	ZEN
Biofarma (Córdoba, Argentina)	5139	HPLC	1				100–250			200–250	2–2.5	20–25	20–25
Romer Labs (Tulln, Austria)	3701	ELISA	1–2				200–250			200–250	2	10–75	20–25
Romer Labs (Union, USA)	3426	LC-MS/MS		1–1.3	1–1.2	1–1.1	100–600	5–100	5–100		0.5–1.1	1–200	1–51.7
Romer Labs (Tulln, Austria)	3106	LC-MS/MS		0.2–1.5	0.2–2.5	0.2–2.5	15–75	5–25	3–25		0.2–7.5	2–25	0.5–25
Biofarma (Córdoba, Argentina)	2351	ELISA	1				250			250			25
Romer Labs (Beijing, China)	2177	ELISA	3	2			200			200	1.9	10	20
Romer Labs (Beijing, China)	1167	LC-MS/MS		0.5	0.5–1	0.5–1	10–20	10	10		0.5	10	5–10
IFA (Tulln, Austria)	696	LC-MS/MS		0.05–1.5	0.057	0.033	1.17–1.5	2.2–4	2.5–4		0.08–1.5	0.14–10	0.07–0.3
Romer Labs (Tulln, Austria)	634	HPLC		0.3	0.1	0.1	50				0.2		10
dsm-firmenich (Shanghai, China)	559	HPLC	2	1	1	1	50	100	100		0.5		10
Romer Labs (Union, USA)	494	ELISA					250			250			40
dsm-firmenich (Madrid, Spain)	400	ELISA	1				200			200	2.5	15	25
Bayrischer Tiergesundheitsdienst (Poing, Germany)	325	ELISA					100						50
dsm-firmenich (Shanghai, China)	321	ELISA	1				250			250		10–25	25
Labocea (France)	285	HPLC	1				15	10	10	20	1	10	10
Romer Labs (Singapore)	268	LC-MS/MS		0.5	0.5–1	0.5–1	10–20	10	10		0.5	10	5–10
Uniwersytet Bydgoszcz (Bydgoszcz, Poland)	95	HPLC					3					0.6	0.2
LUFA (Oldenburg, Germany)	80	ELISA					200						10
BioCheck (Leipzig, Germany)	76	ELISA					50				5	3.5	17
Romer Labs (Union, USA)	65	HPLC		0.7–1.1	0.2–0.9	0.7–0.8	200				0.9		43.1–75
Biomin DE	59	ELISA		1			200–250			200		3.5	17–25
Romer Labs (Singapore)	51	HPLC		0.5–1	0.5–1	0.5–1	50	100	100		0.5		10–32

**Table 2:** Meteorological variables.

Variable Name	Units	Limited Range
Temperature	°C	
Temperature Dew Point	°C	
Relative Humidity	%	[0; 100]
Precipitation Rate	mm/h	
Wind Speed	km/h	
Wind Direction	°	[0; 360]
Surface Pressure	mb	

**Table 3:** Dependency of meteorological variables.

Variable	Source ERA5 Variables	Units
Temperature	2 m temperature	K
Temperature Dew Point	2 m dew point temperature	K
Relative Humidity	2 m dew point temperature	K
Precipitation Rate	Total precipitation	m
Wind Speed	10 m u-component of wind 10 m v-component of wind	m/s m/s
Wind Direction	10 m u-component of wind 10 m v-component of wind	m/s m/s
Surface Pressure	Surface pressure	Pa

**Table 4:** Pre-harvest to post-harvest time frames tested for weather feature aggregation.

Time Frame	Days Before Harvest	Days After Harvest
1	60	0
2	20	10
3	120	60

**Table 5:** Mycotoxins with their associated mycotoxin and fungus groups.

Mycotoxin	Mycotoxin Group	Fungus Group	Threshold (ppb)
Aflatoxin B1	Aflatoxins	*Aspergillus*	2
Aflatoxin B2	Aflatoxins	*Aspergillus*	2
Aflatoxin G1	Aflatoxins	*Aspergillus*	2
Fumonisin B1	Fumonisins	*Fusarium*	500
Fumonisin B2	Fumonisins	*Fusarium*	500
Deoxynivalenol	B-Trichothecenes	*Fusarium*	150
T-2 Toxin	A-Trichothecenes	*Fusarium*	50
Ochratoxin A	Ochratoxin A	*Aspergillus*	10
Zearalenone	Zearalenone	*Fusarium*	50

**Table 6:** Pre-harvest to post-harvest time frame for sample filtering (lab analyzing day).

Time Frame	Days Before Harvest	Days After Harvest
1	15	250

## Data Availability

Data are contained within the article. All derived data are in the Supplementary Materials.

## Supplementary Materials

The following supporting information can be downloaded at: https://www.mdpi.com/article/10.3390/toxins17020077/s1, Figures S1–S6: Line charts depicting mycotoxin levels by country illustrating the relationship between wind direction and mycotoxin concentration; Figure S7: Correlations between weather features; Table S1: Weather trends over 17 years; Table S2: Weather feature labels; Table S3: Weather features to mycotoxins correlations; Read Table S3: Standalone HTML page allowing to read Table S3 as an interactive heatmap.
