# Supplementary material for: Investigating the Correlations Between Weather Factors and Mycotoxin Contamination in Corn: Evidence from Long-Term Data
Source: Toxins (Basel). 2025 Feb 8;17(2):77. doi: 10.3390/toxins17020077 (PMC11861693; doi:10.3390/toxins17020077)
Supplement: Supplementary file 1 [file toxins-17-00077-s001.zip › readTableS3.html]

Interactive Heatmap


# Heatmap of Correlations

Select TableS3.xlsx file:

Don't open Excel at the same time!
  
  

Time Period:

Region:

Country:

Correlation:

Significance:

  
  
