# Supplementary material for: Investigating the Correlations Between Weather Factors and Mycotoxin Contamination in Corn: Evidence from Long-Term Data
Source: Toxins (Basel). 2025 Feb 8;17(2):77. doi: 10.3390/toxins17020077 (PMC11861693; doi:10.3390/toxins17020077)
Supplement: Supplementary file 1 [file toxins-17-00077-s001.zip › supplement.pdf]

Captions for supplement figures and tables for article

Investigating the Correlations Between Weather Factors and Mycotoxin Contamination in Corn: Evidence from Long-Term Data

Figure S1-S6: Line charts depicting mycotoxin levels by country illustrating the relationship between wind direction and mycotoxin concentration. Mycotoxin levels are measured in µg/kg. 'WindDirectionNorthRatio' denotes the proportion of time the wind originated from the North in each country during the period spanning 20 days prior to harvest through 10 days post-harvest; 'WindDirectionSouthRatio' is similarly defined. No significant correlations were observed within individual countries, only with pooling the countries' data the correlations are significant.

Figure S7: Correlations between weather features. Similar to Figure 5, but instead of one representative feature for each strongly correlated weather feature cluster, all weather features are shown. The correction for p-values is also with Bonferroni, but as there are more features, the correction is stricter.

Table S1: weather trends over 17 years. The 'timePeriod' is encoded as before / minus days from harvest and to / after days from harvest, e.g., 'm20_to10' denotes the time from 20 days before harvest to 10 days after harvest. If a country cell is empty, it means all countries of this region. If a region cell is empty, it means all regions. 'PearsonCorr' and 'SpearmanCorr' are quite straightforward these two correlation coefficients. 'OverYearDifferencesSpearmanCorr' is the correlation coefficient of the differences of the WeatherFeature of one year to the year before to the year number. 'minPVsel' is the minimal p-value of that row. Rows are shaded in green to denote significance following a rough correction for multiple testing. The two orange rows are the only significant results directly related to raising temperatures.

Table S2: weather features labels.

Table S3: weather features to mycotoxins correlations. 'All' countries in a row means all countries of that region. The Pearson correlation is just here for information, the corrected p-values are made from the raw Spearman correlation p-values. 'PValueStrict' is the p-value corrected with Bonferroni, 'PValueLoose' is the p-value corrected with FDR. The coloring is made on the two corrected p-values. 'weatherTrendMinPV' is the p-value if that weather feature – the trend of this weather feature in the 17 years of investigation. The 'timePeriod' is encoded as before / minus days from harvest and to / after days from harvest, e.g., 'm20_to10' denotes the time from 20 days before harvest to 10 days after harvest.

Read Table S3: Standalone HTML page allowing to read Table S3 as an interactive heatmap. Available filters are 'timePeriod', 'region' and 'country' columns. Additionally, the correlation measure displayed in the heatmap can be switched between the Pearson and the Spearman correlations. Finally, a filter is available to hide the non-significant correlations on the heatmap.
