# Supplementary figures and images for: Investigating the Correlations Between Weather Factors and Mycotoxin Contamination in Corn: Evidence from Long-Term Data
Source: Toxins (Basel). 2025 Feb 8;17(2):77. doi: 10.3390/toxins17020077 (PMC11861693; doi:10.3390/toxins17020077)

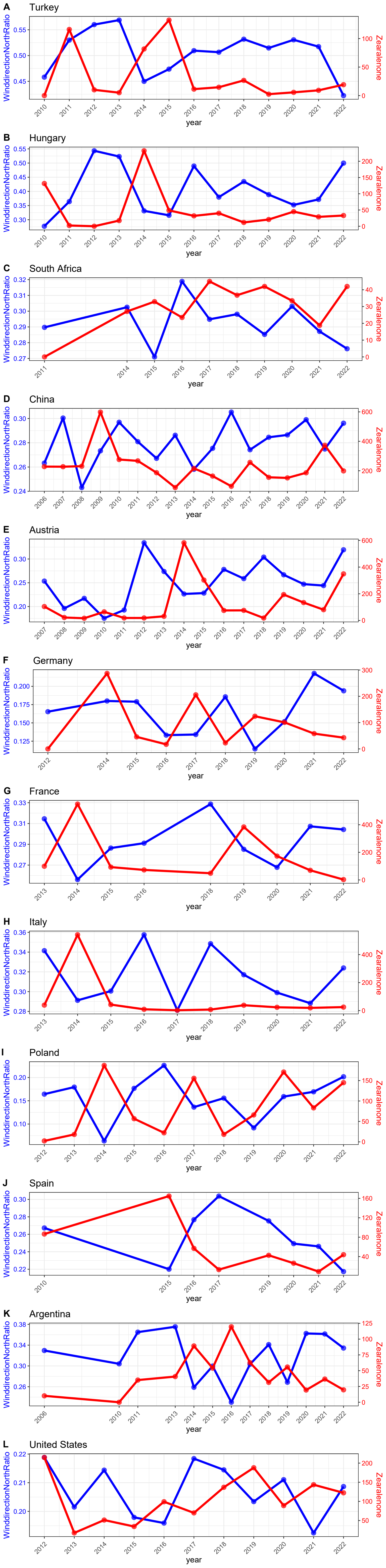

Supplement: Supplementary file 1 [file toxins-17-00077-s001.zip › FigureS1.pdf]

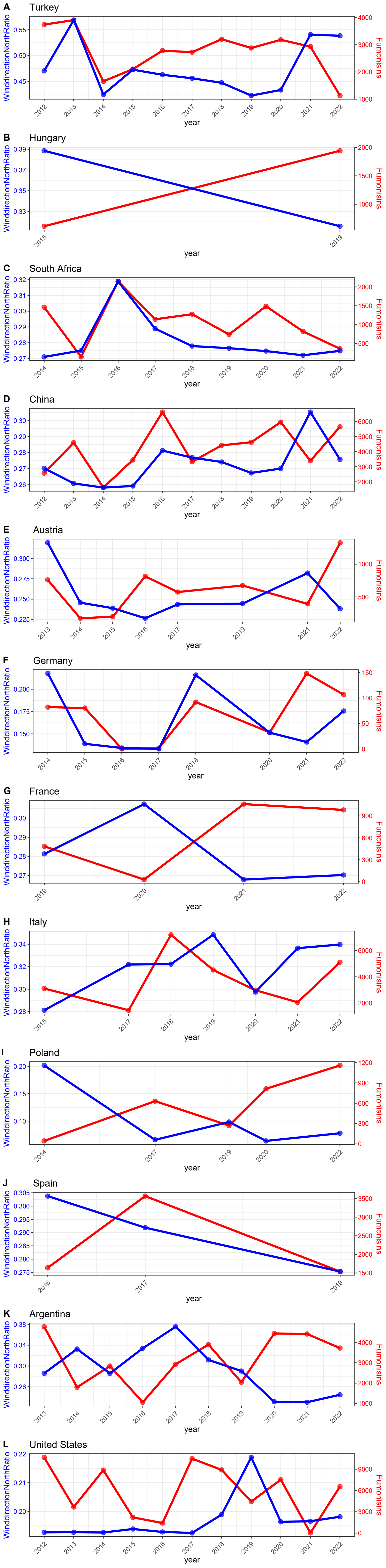

Supplement: Supplementary file 1 [file toxins-17-00077-s001.zip › FigureS2.pdf]

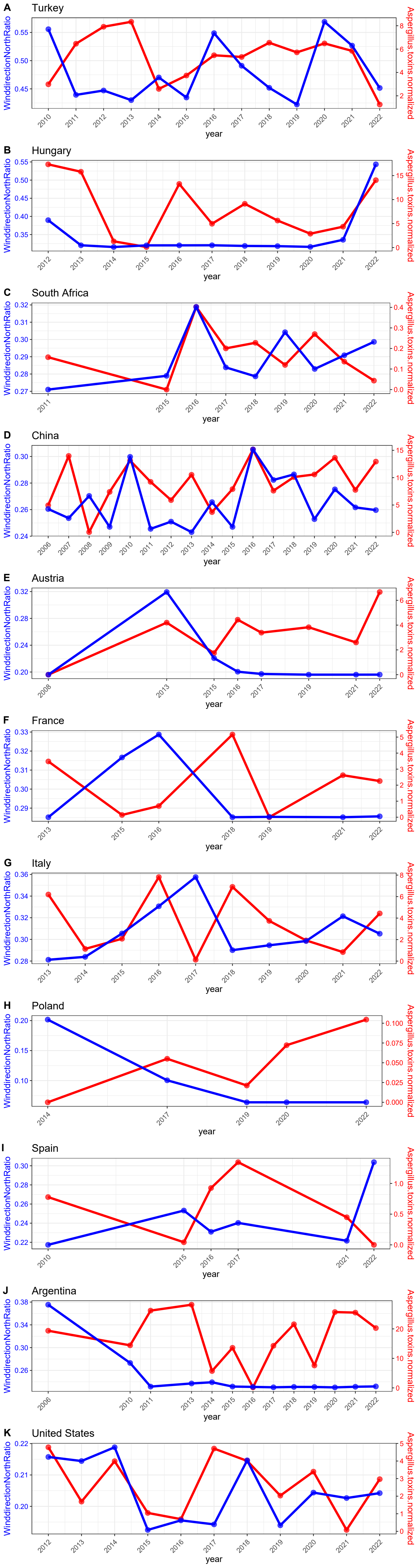

Supplement: Supplementary file 1 [file toxins-17-00077-s001.zip › FigureS3.pdf]

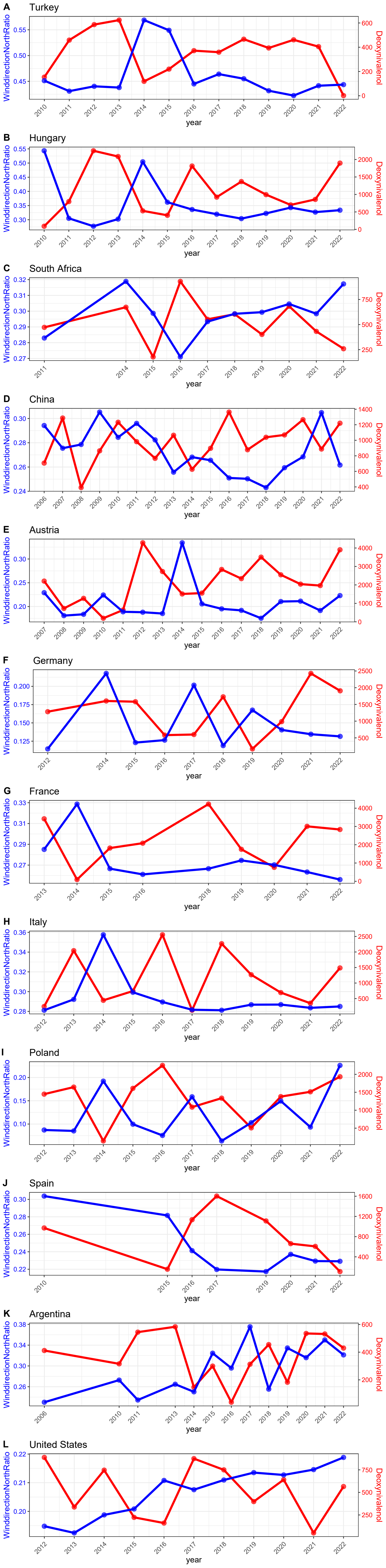

Supplement: Supplementary file 1 [file toxins-17-00077-s001.zip › FigureS4.pdf]

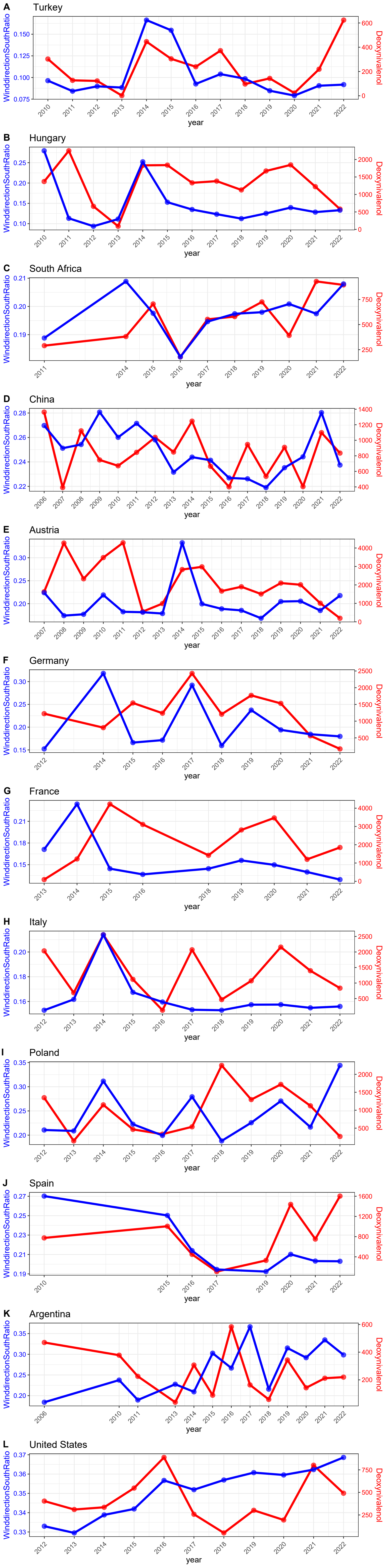

Supplement: Supplementary file 1 [file toxins-17-00077-s001.zip › FigureS5.pdf]

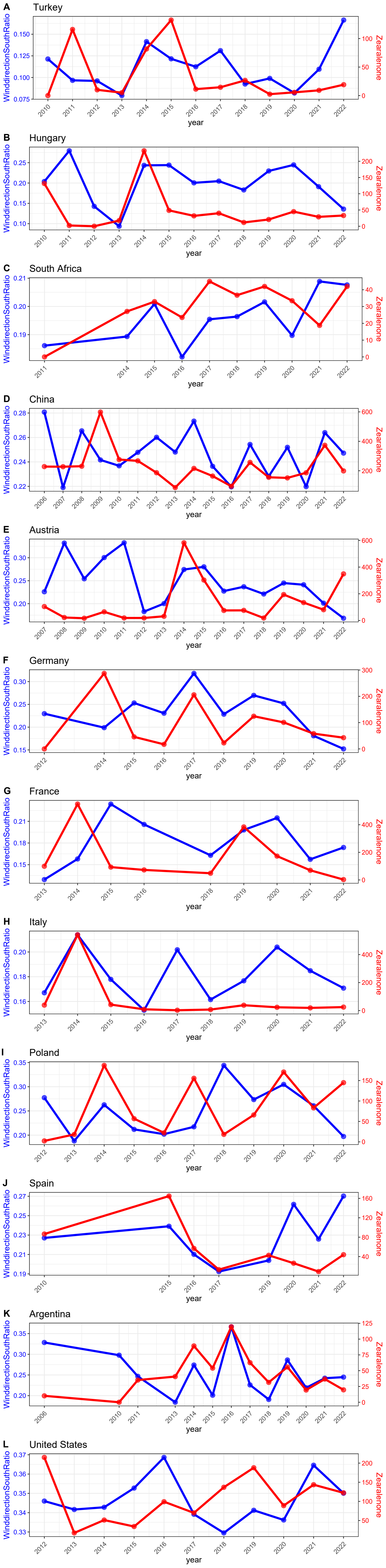

Supplement: Supplementary file 1 [file toxins-17-00077-s001.zip › FigureS6.pdf]

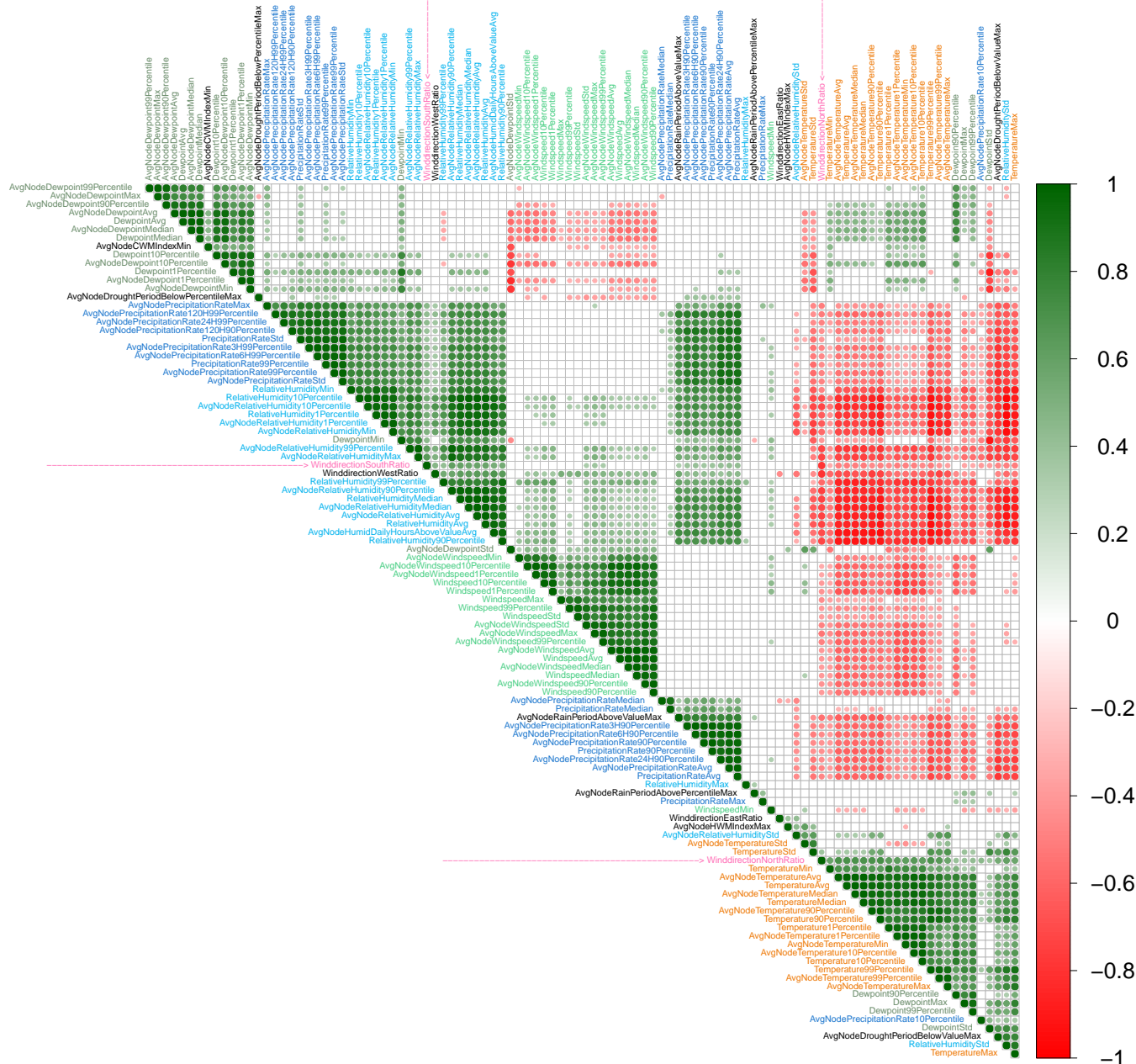

Supplement: Supplementary file 1 [file toxins-17-00077-s001.zip › FigureS7.pdf]
